# Supplementary material for: Patterns of Intron Gain and Loss in Fungi
Source: PLoS Biol. 2004 Nov 30;2(12):e422. doi: 10.1371/journal.pbio.0020422 (PMC532390; doi:10.1371/journal.pbio.0020422)
Supplement: Table S1 — Also available at http://genes.mit.edu/NielsenEtAl/. (4.3 MB ZIP). [file pbio.0020422.st001.zip › NielsenEtAl/html/320.html]

AN8692.1.NCU03151.1.MG02710.1.FG08677.1


```
 CLUSTAL W (1.82) Multiple Sequence Alignments - Introns Inserted


Sequence 1: NCU03151.1	138 aa
Sequence 2: MG02710.1	168 aa
Sequence 3: FG08677.1	166 aa
Sequence 4: AN8692.1	168 aa
Alignment Length: 169 aa
Number Identitical Residues: 55 aa
Alignment Score (without introns) 3163


MG02710.1 	MAALKVGDAFPDGVSFT~YVPTGGKKEDVTA~CGNGIKYNASTE1AKDKKIVIVSVPGAF
NCU03151.1	MSALKAGDSFPE-----~------------A1FAN---------~---KKVVLVAVPGAF
FG08677.1 	MPELKVGADFPEGVFFS~YVQPTPEIAEFST~CGAPTNYNASQE1FKSKKVVLISVPGAF
AN8692.1  	MSGLKAGDSFPADVVFS2YIPWTEEKGEITS~CGIPINYYASKE~WADKKVILFALPGAF
          	*. **.*  ** .   :       .  . ::  .   .  :: .   .**:::.::****

MG02710.1 	TPTCQEQHLKSYVEKKDELKAKGVDKVVFIAY1NDHWVMSAWGKANDIYDDFI~IFASDD
NCU03151.1	TPTCQQQHVPSYLAKLEEFKSKGVDQIIFIAS~NDAWVMAAWGKANGVKDESI0LFMSDA
FG08677.1 	TPTCSASHVPSYVENIEQIKAKGVDQVIVVAV~NDAFVMHGWAKANGITDDKI0LFMSDH
AN8692.1  	TPVCSARHVPEYIERLPEIRAKGVDVVAVLAY~NDAFVMSAWGKANGVKNDDI0LFLSDP
          	**.*.  *: .*: .  ::::**** : .:*  ** :** .*.***.: :: * :* ** 

MG02710.1 	GISFSKSIGWNLGDSGRTARYAIVVDHGKVTYAEKEEAG-GIAVSGAEAVLAKL
NCU03151.1	DCKFSKSIGWTQGD--RTKRYAIVIDHGKVTYAELDEVPKSIANTGADGVLAKL
FG08677.1 	NAKFSTSIGWNLGE--RTGRFAVVIDHGKITYASSDEPG-SIEKSGALGVLPHL
AN8692.1  	EAKFSKSIGWADEE-GRTKRYAIVLDHGKVTYAALEPAKNHLEFSSAETVIKHL
          	  .**.****   : .** *:*:*:****:***  :   . :  :.*  *: :*
```
